# HIV-1 Nef compensates for disorganization of the Immunological Synapse by inducing TGN-associated Lck signaling

**DOI:** 10.1186/1742-4690-8-S2-P22

**Published:** 2011-10-03

**Authors:** Xiaoyu Pan, Jochen M Rudolph, Libin Abraham, Anja Habermann, Claudia Haller, Jacomine Krijnse-Locker, Oliver T Fackler

**Affiliations:** 1 Department of Infectious Diseases, Virology, University Hospital Heidelberg, INF 324, 69120 Heidelberg, Germany

## Background

The Nef protein of human immunodeficiency virus type-1 (HIV-1) facilitates viral replication and disease progression *in vivo.* Nef disturbs the organization of immunological synapses (IS) between infected CD4^+^ T lymphocytes and antigen-presenting B lymphocytes. These effects include the potent retargeting of the T cell receptor (TCR) proximal kinase Lck from the plasma membrane to intracellular membranes. Consequently, Nef interferes with TCR proximal signaling. Paradoxically, Nef enhances distal TCR signaling in infected CD4^+^T lymphocytes, an effect thought to be involved in its role in AIDS pathogenesis.

## Materials and methods

Using live-cell imaging, quantitative confocal microscopy and cell fractionation of Nef-expressing cells and HIV-1-infected primary human T lymphocytes, we analyzed by which mechanism Nef affects the subcellular localization of Lck and addressed the functional consequences of this retargeting for HIV-1 replication.

## Results

We found that Nef induces intracellular compartmentalization of TCR signaling to adjust TCR responses to antigenic stimulation. Nef prevents anterograde transport of newly synthesized pools of the TCR signaling master switch Lck to reroute kinase-active Lck away from the plasma membrane (PM) to the trans-Golgi network (TGN). Via this mechanism, Nef prevents recruitment of active Lck to the IS upon TCR engagement and limits signal initiation at the PM. Instead, Nef triggers Lck-dependent activation of TGN-associated Ras-Erk signaling to promote production of the T lymphocyte survival factor IL-2 and to enhance virus spread. Overexpression of the Lck PM transporter Unc119 restores Nef-induced subversions of Lck trafficking and TCR signaling. Nef thus hijacks Lck sorting to selectively activate TGN-associated arms of compartmentalized TCR signaling.

## Conclusions

These findings reveal a mechanism by which Nef simultaneously restricts proximal and enhances select aspects of distal TCR signaling, respectively. By tailoring T lymphocyte responses to antigenic stimulation, Nef optimizes the environment for HIV-1 replication.

